# Interspecific transfer of parasites following a range‐shift in *Ficedula* flycatchers

**DOI:** 10.1002/ece3.4677

**Published:** 2018-11-11

**Authors:** William Jones, Katarzyna Kulma, Staffan Bensch, Mariusz Cichoń, Anvar Kerimov, Miloš Krist, Toni Laaksonen, Juan Moreno, Pavel Munclinger, Fred M. Slater, Eszter Szöllősi, Marcel E. Visser, Anna Qvarnström

**Affiliations:** ^1^ Department of Animal Ecology, Evolutionary Biology Centre Uppsala University Uppsala Sweden; ^2^ MEMEG, Molecular Ecology and Evolution Group, Department of Biology Lund University Lund Sweden; ^3^ Institute of Environmental Sciences Jagiellonian University Kraków Poland; ^4^ Faculty of Biology M.V. Lomonosov Moscow State University Moscow Russia; ^5^ Department of Zoology and Laboratory of Ornithology, Faculty of Science Palacky University Olomouc Czech Republic; ^6^ Natural Resources Institute Finland (Luke) Turku Finland; ^7^ Section of Ecology, Department of Biology University of Turku Turku Finland; ^8^ Departamento de Ecologia Evolutiva Museo Nacional de Ciencias Naturales (CSIC) Madrid Spain; ^9^ Department of Zoology, Faculty of Science Charles University Prague Czech Republic; ^10^ School of Biosciences Cardiff University Cardiff UK; ^11^ Department of Systematic Zoology and Ecology Eötvös Loránd University Budapest Hungary; ^12^ Department of Animal Ecology Netherlands Institute of Ecology (NIOO‐KNAW) Wageningen The Netherlands

**Keywords:** avian malaria, community ecology, *Ficedula*, parasitology, range expansion

## Abstract

Human‐induced climate change is expected to cause major biotic changes in species distributions and thereby including escalation of novel host‐parasite associations. Closely related host species that come into secondary contact are especially likely to exchange parasites and pathogens. Both the Enemy Release Hypothesis (where invading hosts escape their original parasites) and the Novel Weapon Hypothesis (where invading hosts bring new parasites that have detrimental effects on native hosts) predict that the local host will be most likely to experience a disadvantage. However, few studies evaluate the occurrence of interspecific parasite transfer by performing wide‐scale geographic sampling of pathogen lineages, both within and far from host contact zones. In this study, we investigate how haemosporidian (avian malaria) prevalence and lineage diversity vary in two, closely related species of passerine birds; the pied flycatcher *Ficedula hypoleuca* and the collared flycatcher *F. albicollis* in both allopatry and sympatry. We find that host species is generally a better predictor of parasite diversity than location, but both prevalence and diversity of parasites vary widely among populations of the same bird species. We also find a limited and unidirectional transfer of parasites from pied flycatchers to collared flycatchers in a recent contact zone. This study therefore rejects both the Enemy Release Hypothesis and the Novel Weapon Hypothesis and highlights the complexity and importance of studying host‐parasite relationships in an era of global climate change and species range shifts.

## 1 INTRODUCTION

Global climate change is expected to lead to major shifts in the distribution of species and in their associated parasites and pathogens (Brooks & Hoberg, 2007; Prenter, MacNeil, Dick, & Dunn, 2004). This may have important and complicated effects on whole community structures (Rigaud, Perrot‐Minnot, & Brown, 2010). Rapid range expansion can lead to hosts escaping from their parasites, known as the Enemy Release Hypothesis, and many invasive species therefore experience a competitive advantage over native ones (Menéndez, González‐Megías, Lewis, Shaw, & Thomas, 2008; Phillips et al., 2010; Torchin, Lafferty, Dobson, McKenzie, & Kuris, 2003; Van der Putten, Macel, & Visser, 2010). In a striking example, (Marzal et al., 2011) showed that house sparrows *Passer domesticus* that colonized new areas entirely escaped their natural parasite communities.

A competing theory, the Novel Weapon Hypothesis (NWH), supposes that when hosts are exposed to new parasites or pathogens with which they have no previous evolutionary history, they are expected to pay a relatively high cost of infection (Prenter et al., 2004). Therefore that new parasite‐host associations may be particularly harmful for local naïve communities of (Atkinson & Samuel, 2010; Woodworth et al., 2005). Examples of this include crayfish plague, introduced to several localities around the globe via signal crayfish *Pacifastacus leniusculus,* which has had devastating impacts on local crayfish species (Holdich & Reeve, 1991), or squirrel pox, which was transmitted from the invasive Eastern gray squirrel *Sciurus carolinensis* into the native Eurasian red squirrel *S. vulgaris* populations in Europe (Rushton et al., 2006). New host‐parasite associations are therefore considered to be an increasing concern for both human and wildlife populations and gaining an increased understanding how shifts in host distributions influence interspecific transfer of parasites and pathogens is an urgent matter.

It is important to note that the effects of parasites on their hosts or their ability to switch between hosts may vary across the host's range, either as wide‐scale macro‐differences or as fine‐scale differences across microhabitats (Gandon, 2002; Kaltz & Shykoff, 1998). Despite this, only a few studies have previously attempted to investigate interspecific parasite diversity on a larger (i.e., population‐wide) spatial scale and these studies have found that host species is a better predictor of parasite community than geography (Dubiec et al., 2016; Pulgarín‐R, Gómez, Robinson, Ricklefs, & Cadena, 2018; Scordato & Kardish, 2014). This could mean that host switching is a relatively rare phenomenon that may need a specific set of ecological conditions for the parasite. However, a large‐scale study on haemosporidian parasites in a *Hippolais* warbler hybrid zone in Western Europe found that parasites are not only able to switch between closely related host species, but that this transfer can be asymmetric with the expanding species transferring parasites to the retreating species (Reullier, Pérez‐Tris, Bensch, & Secondi, 2006). In addition, a recent study on parasite diversity in manakin species also includes some sampling in contact zones (Fecchio et al., 2017). However, we are aware of no studies that specifically incorporate wide‐scale sampling of this depth, both within and far from host contact zones in species that directly compete in sympatry.

Here, we investigate how haemosporidian (avian malaria) prevalence and lineage diversity vary in two, closely related species of passerine birds; the collared flycatcher *Ficedula albicollis* and the pied flycatcher *F. hypoleuca* in both allopatry and sympatry.

Collared and pied flycatchers are two closely related, hole‐nesting passerine birds that are common in woodlands across Europe, with a broad overlap in their range in Central Europe and on some of the Baltic islands (Alatalo, Gustafsson, & Lundberg, 1994; Sætre, Kral, Bures, & Ims, 1999). These two species are closely related with a high degree of physiological similarity making them likely to be compatible hosts for an array of parasites (Kulma, Low, Bensch, & Qvarnström, 2013). Both species are migrants, spending the nonbreeding season in woodland savanna habitats in separate areas of sub‐Saharan Africa (see map in Veen et al., 2014). The two species probably diverged in allopatry, in the glacial refugia of southern Europe, roughly one million years ago and have subsequently come into secondary contact since the last glacial maximum (Nadachowska‐Brzyska et al., 2013; Sætre et al., 2001). The contact zone is dynamic, with collared flycatchers naturally encroaching northward (Huntley, Green, Collingham, & Willis, 2007), including the Swedish island of Öland, which was colonized about 60 years ago (Qvarnström, Wiley, Svedin, & Vallin, 2009). Both species commence breeding from early May to mid June, with more southerly and westerly populations tending to breeding earlier than northern‐most populations (Sanz, 1997; Sirkiä et al., 2018).

Haemosporidian parasites, more commonly referred to as avian malaria, are a diverse group of apicomplexan protists that include two commonly studied genera (*Haemoproteus* and *Plasmodium*) (Clark, Clegg, & Lima, 2014). Haemosporidians are an ideal parasite group to investigate as their life‐history traits and species and lineage diversities are well studied and documented (Bensch, Hellgren, & Pérez‐Tris, 2009). In addition, they are widely distributed around the world with each continent having distinct communities (Clark, 2018; Ricklefs, Fallon, & Bermingham, 2004). All haemosporidians require a vertebrate host and an insect vector to complete their life cycle, particularly *Culex* mosquitoes and *Culicoides* biting midges, which are the most relevant insect vectors in this system (Glaizot et al., 2012; Tomás et al., 2008).

*Ficedula* flycatchers are an ideal system to study the interaction between parasites and different host species living in sympatry due to their recent range shifts as a result of climate change (Sætre et al., 2001). In addition, their wide and partially overlapping breeding distributions and wholly allopatric wintering distributions allow us to investigate parasite communities in allopatry, in sympatry and at a naturally occurring range expansion. A previous study of haemosporidians in a Swedish population of flycatchers found possible evidence for the Novel Weapon Hypothesis as collared flycatchers do comparatively better than pied flycatchers when exposed to the same parasites (Kulma et al., 2013). However, a formal survey of parasites in sympatric and allopatric populations has yet to be conducted to further test this idea.

In this study, we investigate how parasite prevalence and diversity vary spatially across the breeding ranges of the two flycatcher species. We also investigate whether flycatcher populations in sympatry have higher similarity in parasite community composition than flycatcher populations in allopatry. We predict that if the “escape” hypothesis is supported, collared flycatchers in the northern invasion front will have lower parasite prevalence than collared flycatchers in the core range. We also predict that pied and collared flycatchers will share more European lineages of malaria than African lineages, due to their completely allopatric wintering distributions and that parasite transfer should be most likely to occur from collared to pied flycatchers.

## 2 MATERIALS AND METHODS

**Figure 1:**
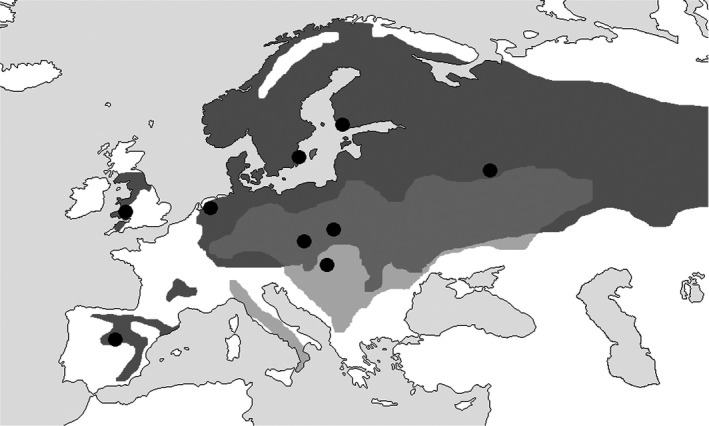
Map showing the breeding distributions of pied (dark gray) and collared flycatchers (light gray). The overlap zone is denoted with midgray. Sampling locations are marked with black circles. Map adapted from (Qvarnström, Rice, & Ellegren, 2010)

Samples were collected from nine locations across the European breeding ranges of both pied and collared flycatchers (Figure 1 and Table 1). In total, 1,503 blood samples were collected during the breeding seasons between 2004 and 2011 from adult birds. DNA was extracted in a variety of methods depending on the storage medium. DNA concentration was quantified using a NanoDrop2000 (Thermo Scientific). To ascertain infection status, samples were screened in the same laboratory in Uppsala, Sweden, for *Plasmodium* and *Haemoproteus* presence using an established nested PCR technique, which amplifies a 478 bp region of the cyt b gene (Waldenström, Bensch, Hasselquist, & Östman, 2004). Negative (ddH2O) and positive controls (samples from birds previously confirmed to be infected) were included to control for possible contaminations and amplification failures during PCRs, respectively. PCR products were then stained with GelGreen and visually inspected for haemosporidian presence or absence on 2% agarose gel. Positive samples were sent to Macrogen Inc., Seoul, for sequencing. The resulting sequences were then aligned and compared with MEGA7© software to available sequences in the MalAvi database (Bensch et al., 2009). Transmission area of a lineage was ascertained from MalAvi by assessing the distributions of other host species infected by that lineage. For example, pLAMPUR03 was assigned as an African lineage as it had also been discovered in the purple‐headed starling *Hylopsar purpureiceps* which is a resident species in Central Africa. Some lineages were only found in *Ficedula* flycatchers or in other migratory species and were therefore designated as “Unknown”.

**Table 1:** Details from the nine sampling locations included in this study, including sample sizes, sampling period, haemosporidian prevalence and diversity (Shannon Diversity Index)

Species	Country	Site	Sampling period	Sample size	Infection rate	Lineages	Diversity
CF	Czechia	Stařechovice, Olomouc	2007, 2009	96	21.9	5	1.33
PF	Finland	Hirvensalo, Turku	2011	80	91.3	8	1.27
CF	Hungary	Pilis Mountains	2004, 2005	117	30.8	16	2.51
PF	Netherlands	Hoge Veluwe NP, Gelderland	2009, 2010	60	63.3	4	0.48
CF	Poland	Drwinia, Bochnia	2005	67	34.3	8	1.8
PF	Russia	Zvenigorod, Moscow	2007, 2011	169	61.5	10	1.46
PF	Spain	Valsaín, Castilla y León	2010, 2011	192	51.0	12	1.73
CF	Sweden	Öland, Kalmar Län	2004, 2005	365	32.1	21	2.15
PF	Sweden	Öland, Kalmar Län	2004, 2005	168	48.2	8	1.41
PF	UK	Llanwrthwl, Powys	2004, 2005	189	31.2	10	1.54

### 2.1 Statistical methods

Parasite diversity was analyzed using the Shannon diversity index, which accounts for species richness, abundance, and evenness (Peet, 1975). Species richness was also separately analyzed to account for sample size differences between the populations (packages vegan; rarefy). Population community structure was assessed by using nonmetric multidimensional scaling (NMDS) models to ascertain overall differences between collared and pied flycatcher populations, as well as differences in transmission location and parasite genus. Differences in community structure between collared and pied flycatchers were assessed with MANOVAs. Finally, spatial community similarity was checked with a permutational analysis of variance using distance matrices (ADONIS) using 100,000 permutations based on Jaccard distances (package vegan). In addition, the effect of geographic distance on population community relatedness was carried out with a partial‐Mantel test (package adegenet) with 1,000,000 permutations using distance matrices of lineage prevalences and geographic distance between sampling populations. Statistical analyses were carried out using the software R (version 3.4.2). The maximum‐likelihood phylogenetic tree was created using the software MEGA7©. Topology robustness was assessed with 1,000 bootstraps.

## 3 RESULTS

**Figure 2:**
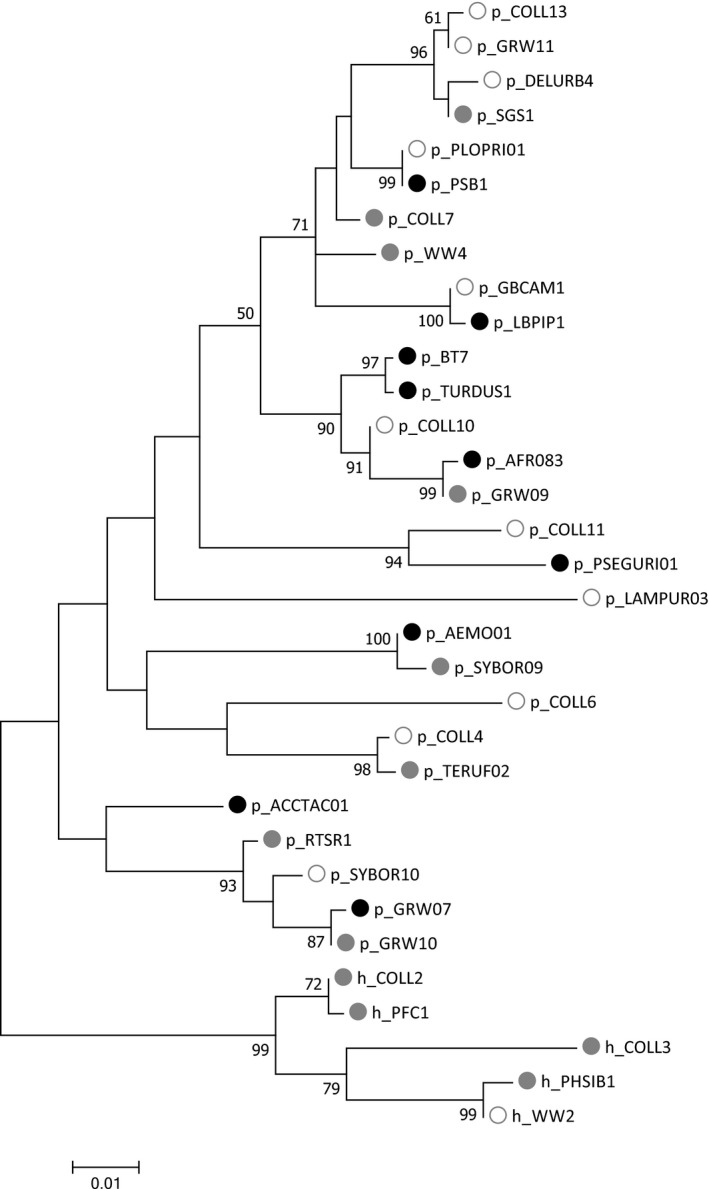
Maximum‐likelihood phylogenetic relationships of the 33 haemosporidian lineages detected in this study (5 *Haemoproteus* and 28 *Plasmodium* lineages). Black circles indicate lineage‐host specificity within the bounds of this study (black = pied flycatcher, white = collared flycatcher, and gray = both species). The phylogenetic tree was constructed from partial cytochrome b sequences (479 bp). Node labels represent bootstrap values (1,000 replicates)

**Figure 3:**
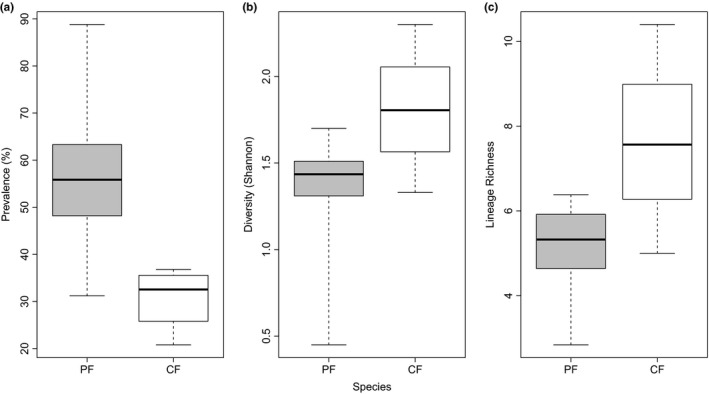
Boxplots showing (a) haemosporidian prevalence; (b) haemosporidian diversity (Shannon Index) and (c) haemosporidian lineage richness in pied (PF) and collared (CF) flycatchers

**Figure 4:**
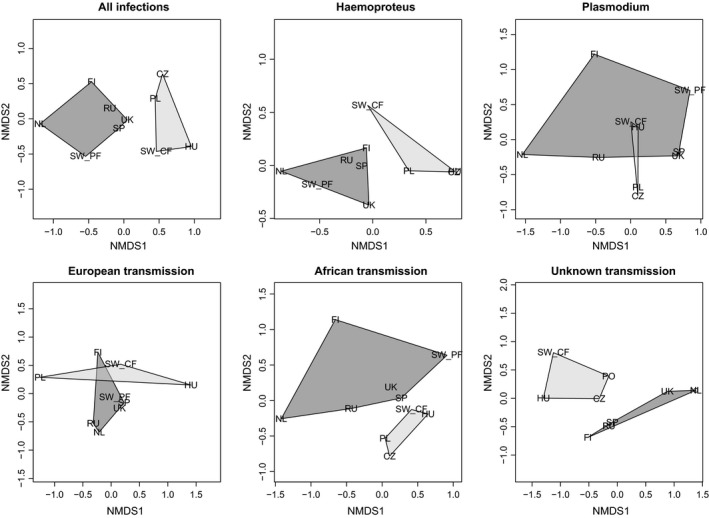
Non‐metric multidimensional scaling (NMDS) plots of haemosporidian parasite communities (All infections, *Haemoproteus, Plasmodium,* African transmission, European transmission, and unknown transmission locations). Dark polygons represent pied flycatcher populations while pale polygons represent collared flycatcher populations

In total 5 *Haemoproteus* and 28 *Plasmodium* lineages were detected in the entire dataset. All infections, but one (pCOLL13) could be assigned to a previously published lineage in the MalAvi database. In addition, three samples were found to contain mixed infections and five samples were unable to be sequenced successfully; these samples were included in prevalence analyses, but excluded from all other analyses. Flycatcher populations varied widely in their overall parasite community compositions with only three (hPHSIB1, hCOLL2, hPFC1) and four (pRTSR1, pCOLL7, hCOLL2, hCOLL3) lineages being detected in all pied and collared flycatcher populations, respectively; and only one lineage (hCOLL2) was found in both species and all seven populations (see Table 1 and Supporting information: Table S1). hPFC1 was the most abundant strain in five of the seven populations of pied flycatchers and the second most abundant in the remaining two. hPHSIB1 was the most abundant strain in Swedish collared flycatchers, but rare or absent in the Central European populations. For a full list of lineage names and prevalences, see the Supporting information: Table S1. A maximum‐likelihood phylogeny of all detected lineages shows that there is no relationship between lineage relatedness and host specificity in this study (Figure 2). T tests showed that overall prevalence was significantly higher in pied flycatcher populations than in collared flycatcher populations (*df* = 6.754*, p = *0.019). By contrast, parasite lineage richness and diversity tended to be higher in collared flycatcher populations (Figure 3) although this was not statistically significantly higher (Shannon diversity: *df* = 7.077,* p = *0.101; species richness: *df* = 4.318, *p = *0.098). In addition, ADONIS analyses showed that breeding site latitude and longitude were not significant predictors of prevalence or diversity for either all infections (latitude: *R* ^2^ = 0.098,* p* = 0.251; longitude: *R* ^2^ *= *0.068,* p* = 0.548) or for European‐transmitted infections (latitude: *R* ^2^ *= *0.128,* p = *0.473; longitude: *R* ^2^ = 0.073, *p = *0.831). Partial‐Mantel tests confirm that geographic location has no significant effect on parasite community structure (*r = *0.144,* p = *0.386). MANOVA tests of NMDS scores, weighted by sample size, of all lineages show that host species is a significant predictor of parasite community structure (Table 2). However, “species” was not a significant predictor of parasite community structure for European‐transmitted lineages and *Plasmodium* infections. Instead, African‐transmitted lineages, lineages of unknown transmission location and *Haemoproteus* infections were significantly predicted by host species (Figure 4, Table 2).

**Table 2:** Multivariate analysis of variance (MANOVA) of Non‐metric multidimensional scaling (NMDS) scores

Parasite	Pillai's trace	*F*	*p*
All infections	0.667	7.031	0.021*
Genus			
*Haemoproteus*	0.644	6.333	0.027*
*Plasmodium*	0.036	0.132	0.879
Transmission			
Africa	0.616	5.619	0.035*
Europe	0.302	1.301	0.339
Unknown	0.763	11.274	0.006**

Statistically significant values are highlighted in bold *p* ≤ .05 (*) and *p* ≤ .01 (**).

## 4 DISCUSSION

We investigated prevalence and lineage diversity of *Plasmodium* and *Haemoproteus* across the European breeding range of collared and pied flycatchers and found evidence consistent with interspecific transfer of parasites following a recent range‐shift of collared flycatchers. We predicted that collared flycatchers in the newly colonized Swedish population would have lower parasite prevalence and diversity but did not find any evidence consistent with collared flycatchers having escaped blood parasites during their recent expansion and range‐shift to the North. By contrast, they have rather gained new parasite lineages as collared flycatchers in recent secondary contact with pied flycatchers were more pied flycatcher‐like in their community structure of *Haemoproteus* lineages. In addition, we found that host species is not a significant predictor of parasite diversity for European‐transmitted lineages, but it is for African lineages and lineages of unknown transmission origin. We also report large variation in prevalence of malaria parasites among pied flycatcher populations that are not explained by longitude or latitude. We further discuss these findings in relation to biotic factors and parasite transmission below.

The overall infection rate was significantly different between the host species, with pied flycatchers having higher infection rates than collared flycatchers. However, pied flycatchers also tended to have a lower diversity of malaria lineages than collared flycatchers. There was also a large variation in infection rates among pied flycatcher populations, with pied flycatchers sampled in Finland having an extremely high infection rate (91.3%), which is the highest recorded infection rate for pied flycatchers published, whereas pied flycatchers sampled in the United Kingdom had the lowest recorded infection rate (31.2%). Differences in infection rates within and between the two host species could be due to multiple factors. It is possible that flycatcher populations are locally adapted to the parasite communities and that there is variation in the diversity of their MHC genes (Bonneaud, Pérez‐Tris, Federici, Chastel, & Sorci, 2006; Westerdahl, 2007). It is also possible that collared and pied flycatchers are exposed to different pressures of ecological immunity and invest into different defense strategies. Cases similar to this have been documented in *Microcebus* mouse lemurs that live sympatrically and in ecologically similar habitats in Madagascar which have divergent MHC alleles to deal with the same nematode infections (Schwensow, Dausmann, Eberle, Fietz, & Sommer, 2010). In addition, collared and pied flycatchers could invest in different aspects of immunity such as resistance versus tolerance (Roy & Kirchner, 2000). The utilization of resistance or tolerance has been tested experimentally with mouse strains infected with the malaria parasite *Plasmodium chabaudi* that showed mouse strains which had higher resistance to the parasite also had a lower tolerance (Råberg, Sim, & Read, 2007). Variation in ecological immunity strategies can also be broadened by the effects of climate and temperature which have been shown to either impede or promote an organism's immune response, depending on the species involved (Garvin, Abroe, Pedersen, Dunn, & Whittingham, 2006; Thomas & Blanford, 2003). This could explain at least part of the observed variation in infection rates and diversity among pied flycatcher populations that are not explained by either longitude or latitude. Further studies are needed to explore the role of MHC diversity, isolation by distance and gene flow between populations in explaining the intraspecific differences in parasite communities.

An alternative, nonmutually exclusive explanation could be that differences in microhabitat and niche use across the breeding ranges of the two species coincide with differences in insect vector communities and density, leading to the infection rates observed in pied flycatchers being higher. Several studies have shown that malaria prevalence can vary on both a large (Durrant et al., 2007; Galen & Witt, 2014) and a small spatial scale (Szöllősi et al., 2011; Wood et al., 2007). In addition, proximity to features such as water bodies or damp ground can affect vector community and density. Areas near standing water are often important for determining prevalence of *Plasmodium*, which is most frequently mosquito‐borne (Wood et al., 2007), whereas damp and marshy ground is more important for parasites vectored by *Culicoides* midges, the primary vectors of *Haemoproteus* (Kirkeby, Bødker, Stockmarr, & Enøe, 2009). However, a recent study of Amazonian birds did not find such a relationship (Pulgarín‐R et al., 2018). Taken together, we therefore theorize that microhabitat differences largely explain the differences in infection rate observed between populations, while different immune responses may dictate interspecific differences in infection rate.

Despite the fact that pied flycatchers have a significantly higher rate of blood parasite infections, the pattern of haemosporidian lineage diversity goes in the opposite direction. There is a nonsignificant trend for higher diversity of haemosporidian lineages in collared flycatchers than in pied flycatchers. This tendency for higher diversity may be driven by rarer, African‐transmitted lineage*s*. Possibly the high diversity of African lineage*s* found in collared flycatchers and the significant difference in African‐transmitted lineages between the two species is associated with the different climatic conditions they encounter during the winter. While both flycatcher species occupy the same habitat types during the nonbreeding season: woodland savannah and gallery forest; collared flycatchers winter entirely south of the equator, and therefore, encounter higher rainfall than pied flycatcher populations wintering north of the equator. Insect vectors that carry *Plasmodium* lineages (which make up the majority of African lineages in this study) are known to require standing water or wet ground to breed, and therefore, become more abundant during the wet seasons (Cosgrove, Wood, Day, & Sheldon, 2008; Craig, Le Sueur, & Snow, 1999). Higher vector abundance, in turn, increases the rate of infection in secondary host species such as birds. This is also demonstrated by patterns of human malaria infections, which are generally higher during the wet season (Craig et al., 1999). In addition, African lineage diversity could be more likely to be different in the two host species due to the large distance between their allopatric distributions and documented differences in parasite diversity (Loiseau et al., 2012).

We found that the two bird host species had more similar parasite diversities in sympatry than in allopatric populations (Figure 4). This finding was mostly driven by the presence of two European‐transmitted *Haemoproteus* lineages in the Swedish collared flycatcher population that were abundant in all pied flycatcher populations, but rare or completely absent in the other collared flycatcher populations (hPFC1 & hPHSIB1). hPFC1 appears to be a specialist of pied flycatchers and was found in low levels in the Swedish collared flycatcher population in our study (3.4%). It is interesting to note that the lineage is abundant in a population of pied flycatchers in the Central European contact zone in Poland (Dubiec, Podmokła, Harnist, & Mazgajski, 2017)) and is absent in the Polish collared flycatchers in our study. However, it is possible that we have failed to detect this lineage in our relatively small sample size (*n* = 67) of collared flycatchers from Poland. hPFC1 has also recently been detected in extremely low levels in collared flycatchers from Hungary, in a much more extensively screened population of birds, which suggests that it may occasionally find a collared flycatcher host in Central Europe (Szöllősi et al., 2016). hPHSIB1 appears to have a broader host range; also being present in 15 other passerine species. It is important to note that two of our populations of collared flycatchers (Czechia and Poland) lie in the historical contact zone between pied and collared flycatchers and while these populations are not directly sympatric with pied flycatchers, it is possible that they can be found in nearby woodlands (Dubiec et al., 2017)).

Previous studies have found evidence for asymmetrical transfer of parasites from invading, more competitive hosts, which are in the process of expanding their distribution ranges, to native hosts (Beadell et al., 2006; Reullier et al., 2006; Shea & Chesson, 2002). By contrast we have shown that collared flycatchers, that is, the more competitive species that are in the process of expanding their distribution range in response to a warming climate (Kulma et al., 2013; Sirkiä et al., 2018), appear to acquire new malaria lineages from the native pied flycatchers. In addition, collared flycatchers have maintained the high diversity of species‐specific malaria lineages in the newly colonized areas in Sweden and have not been able to escape the parasites in their core range. Therefore, we can reject the Enemy Release Hypothesis in this system.

While there are some differences in parasite communities and some evidence for host switching, it is important to assess whether these lineages play a role in the competitive dynamics observed between the two *Ficedula* species. One of the lineages that appears to be transferred from pied flycatchers to collared flycatchers, hPHSIB1, has been found to have negligible effects on survival or lifetime reproductive success of female collared flycatchers (Kulma, Low, Bensch, & Qvarnström, 2014). By contrast, pied flycatcher females seem to experience reduced survival when infected with this lineage (Kulma et al., 2013) despite the fact that this species probably share a longer coevolutionary history with the parasite. However, the fact that parasite communities in Swedish pied flycatchers are not different from allopatric populations suggests that we must reject the Novel Weapon Hypothesis in this system too. Collared flycatchers hence appear to be better able to deal with *Haemoproteus* lineages in general when compared to pied flycatchers, possible due to higher overall exposure to other more virulent haemosporidian lineages (e.g., due to differences in overwinter grounds) in their recent evolutionary history. The finding that collared flycatchers carry a higher diversity of haemosporidian lineages than pied flycatchers is indeed consistent with a possible higher exposure to virulent lineages.

In order to fully understand how shared *Haemoproteus* lineages influence competitive interactions between the two flycatcher species, we need to further investigate differences in immunological characteristics of the two species and further evaluate the role of nonshared lineages in selection favouring such possible differences in immunological characteristics between the two species. Our findings highlight the complexity of host‐switching dynamics in an era of changes in species distributions related to increased habitat disturbances, human‐induced species introductions and global climate change.

## AUTHOR CONTRIBUTIONS

WJ, KK, and AQ designed the study; MC, AK, MK, TL, JM, FMS, ES, MEV, and AQ provided samples; WJ, KK, PM, and ES carried out lab work; SB provided assistance with the phylogenetic tree; WJ analyzed the data; WJ and AQ wrote the paper; all authors provided comments and suggestions. The authors declare no conflict of interest.

## DATA ACCESSIBILITY

Lineage data has been submitted to the public MalAvi Database. Additional data has been submitted to Dryad https://doi.org/10.5061/dryad.7t4s040. Parasite lineage information has been deposited in the MalAvi database. Sequence data from COLL13 will be deposited in GenBank.
