# Supplementary material for: Interspecific transfer of parasites following a range‐shift in Ficedula flycatchers
Source: Ecol Evol. 2018 Nov 11;8(23):12183–92. doi: 10.1002/ece3.4677 (PMC6303764; doi:10.1002/ece3.4677)
Supplement: Supplementary file 1 [file ECE3-8-12183-s001.docx]

Supplementary material

Table S1 showing the prevalence of malaria lineages in each flycatcher population

|  | ***F. hypoleuca*** | | | | | | ***F. albicollis*** | | | |
| --- | --- | --- | --- | --- | --- | --- | --- | --- | --- | --- |
|  | **Finland** | **Netherlands** | **Russia** | **Spain** | **SwedenPF** | **UK** | **Czechia** | **Hungary** | **Poland** | **SwedenCF** |
| ***Plasmodium*** | | | | | | | | | | |
| **ACCTAC01** |  |  |  |  |  | 1 |  |  |  |  |
| **RTSR1** |  |  | 3 | 2 |  | 2 | 2 | 3 | 3 | 3 |
| **GRW07** |  |  |  |  |  |  |  |  |  | 1 |
| **GRW10** |  | 1 | 2 |  |  |  |  |  |  | 1 |
| **SYBOR10** |  |  |  |  | 2 |  |  |  |  | 2 |
| **AEMO01** |  |  |  |  |  | 1 |  |  |  |  |
| **SYBOR09** |  |  |  |  |  |  |  | 1 |  |  |
| **COLL6** |  |  |  |  |  |  | 1 |  | 1 | 2 |
| **COLL4** |  |  |  |  |  |  |  | 1 |  | 2 |
| **TERUF02** |  |  | 1 |  |  |  |  |  |  | 1 |
| **COLL11** |  |  |  |  |  |  |  | 1 |  | 1 |
| **PSEGRI01** | 1 |  |  |  |  |  |  |  |  |  |
| **LAMPUR03** |  |  |  |  |  |  |  | 1 | 1 | 1 |
| **BT7** | 1 |  |  |  |  |  |  |  |  |  |
| **TURDUS1** | 1 |  |  |  |  |  |  |  |  | 1 |
| **COLL10** |  |  |  |  |  |  |  | 1 |  | 1 |
| **AFR083** |  |  | 1 |  |  |  |  |  |  |  |
| **GRW09** | 1 |  | 11 | 2 | 2 | 3 |  | 5 | 2 | 4 |
| **WW4** |  |  |  | 1 | 1 |  |  |  |  | 2 |
| **GBCAM1** |  |  |  |  |  |  |  | 1 |  |  |
| **LBPIP1** |  |  |  |  | 1 |  |  |  |  |  |
| **GRW11** |  |  |  | 3 |  |  |  |  |  |  |
| **DELURB4** |  |  |  |  |  |  |  | 1 |  |  |
| **SGS1** |  |  |  | 2 | 4 | 1 |  | 1 |  | 3 |
| **COLL7** |  |  |  | 1 |  |  | 2 | 1 | 1 | 3 |
| **PLOPRI01** |  |  |  |  |  |  |  | 1 |  |  |
| **PSB1** |  |  | 1 | 4 |  | 3 |  |  |  |  |
| **COLL13** |  |  |  |  |  |  |  | 1 |  |  |
| ***Haemoproteus*** | | | | | | | | | | |
| **PHSIB1** | 30 | 1 | 13 | 21 | 24 | 2 |  |  | 5 | 66 |
| **WW2** |  |  |  |  |  |  |  |  |  | 1 |
| **COLL3** | 6 |  | 5 | 7 |  | 1 | 6 | 8 | 2 | 5 |
| **COLL2** | 3 | 2 | 5 | 11 | 12 | 29 | 9 | 7 | 8 | 12 |
| **PFC1** | 28 | 34 | 61 | 43 | 35 | 16 |  |  |  | 4 |
| **Unidentified** | | | | | | | | | | |
|  | 2 |  |  | 1 |  |  | 1 | 2 |  | 1 |
